# Safety assessment of sorafenib in Chinese patients with unresectable hepatocellular carcinoma: subgroup analysis of the GIDEON study

**DOI:** 10.1186/s12885-018-4144-9

**Published:** 2018-03-02

**Authors:** Sheng-Long Ye, Jiamei Yang, Ping Bie, Shuijun Zhang, Xiaoping Chen, Fengyong Liu, Luming Liu, Jie Zhou, Kefeng Dou, Chunyi Hao, Guoliang Shao, Qiang Xia, Yajin Chen, Jijin Yang, Xiaxing Deng, Yunpeng Liu, Yunfei Yuan, Zhiren Fu, Keiko Nakajima, Zhengguang Lv

**Affiliations:** 1 Liver Cancer Institute, Zhongshan Hospital, Fudan University, 136 Yixueyuan Rd, Shanghai, 200032 China; 2 Department of Special Treatment, Eastern Hepatobiliary Surgery Hospital, Shanghai, China; 3 Institute of Hepatobiliary Surgery, Southwest Hospital, Third Military Medical University, Chongqing, China; 4 Department of General Surgery, The First Affiliated Hospital of Zhengzhou University, Zhengzhou, China; 5 Department of Surgery, Tongji Medical College, Huazhong University of Science and Technology, Wuhan, China; 6 Department of Interventional Radiology, Chinese PLA General Hospital, Beijing, China; 7 Shanghai Cancer Center, Fudan University, Shanghai, China; 8 Department of Hepatobiliary Surgery, Nanfang Hospital of Southern Medical University, Guangzhou, China; 9 Department of Hepatobiliary Surgery, Xijing Hospital, Xi’an, China; 10 Department of Hepato-Pancreato-Biliary Surgery, Beijing Cancer Hospital, Peking University, Beijing, China; 11 Department of Radiology, Zhejiang Cancer Hospital, Hangzhou, China; 12 Department of Liver Surgery, Renji Hospital, Shanghai Jiaotong University School of Medicine, Shanghai, China; 13 Department of Hepatobiliary Surgery, Second Affiliated Hospital of Sun Yat-Sen University, Guangzhou, China; 14 Department of Nuclear Medicine, Changhai Hospital, Second Military Medical University, Shanghai, China; 15 Department of General Surgery, Ruijin Hospital, Shanghai Jiaotong University School of Medicine, Shanghai, China; 16 Department of Medical Oncology, the First Hospital of China Medical University, Shenyang, China; 17 Department of Hepatobiliary, Cancer Center, Sun Yat-sen University, Guangzhou, China; 18 Department of Liver Transplantation, Shanghai Changzheng Hospital, Shanghai, China; 19 Bayer Healthcare Pharmaceuticals, Whippany, NJ USA; 20 Bayer Healthcare Company Ltd., Beijing, China

## Abstract

**Background:**

This study aimed to investigate the safety of sorafenib for the treatment of unresectable hepatocellular carcinoma in Chinese patients.

**Methods:**

A subgroup of 345 Chinese patients from the international database of the Global Investigation of therapeutic DEcisions in hepatocellular carcinoma and Of its treatment with sorafeNib (GIDEON) study was included in this analysis. Safety assessment measures were adverse events (AEs) and serious adverse events (SAEs) graded using the National Cancer Institute Common Terminology Criteria version 3.0.

**Results:**

Of 331 evaluable patients, 98% started sorafenib at 800 mg/day. The median treatment duration was 22 weeks (range, 0.1–116 weeks), and median overall survival (OS) was 322 days (10.7 months). Approximately 50% of patients had at least one adverse event, and 6% had grade 3–4 adverse events. Drug-related adverse events were experienced by 29% of patients, and 3.6% had grade 3–4 drug-related adverse events. Overall, 23% of patients (*n* = 77) experienced serious adverse events, among which only 1 event was drug-related (0.3%). No differences in overall adverse events, serious adverse events, and deaths were observed between Child-Pugh A and Child-Pugh B patients. The most frequent drug-related adverse events were dermatological/skin (24%), hand-foot skin reaction (20%), gastrointestinal (11%), and diarrhea (11%). The majority of adverse events occurred within 30 days of beginning sorafenib.

**Conclusion:**

Sorafenib has satisfactory efficacy and safety in Chinese Child-Pugh A and B patients with unresectable HCC using the recommended dosage of 800 mg/day, and the safety of sorafenib is not affected by liver function. Prophylaxis for gastrointestinal adverse events may help to decrease dose interruptions or discontinuation.

**Trial registration:**

ClinicalTrials.gov; Identifier: NCT00812175. Date of registration: December 19, 2008.

**Electronic supplementary material:**

The online version of this article (10.1186/s12885-018-4144-9) contains supplementary material, which is available to authorized users.

## Background

Hepatocellular carcinoma (HCC) is the second leading cause of cancer-related deaths worldwide, and the incidence is increasing with 782,000 cases diagnosed worldwide in 2012 [1]. The incidence of HCC is highest in the East and South-East Asia, with China accounting for nearly half of all HCC cases and deaths globally [2]. In Asian countries, the main HCC treatment options include surgical resection, ablation, transarterial chemoembolization (TACE), and radiation or chemotherapy based on liver function [3, 4]. Surgical resection may offer a 5-year survival up to 70% in patients with excellent liver function but for patients with advanced disease, surgical resection is an option for less than 20% of patients [5]. While TACE may provide a meaningful increase in overall survival (OS) for some patients with intermediate (unresectable) disease, many patients in the Asia-Pacific region present with advanced HCC for which most therapies do not provide a meaningful increase in OS [6].

Sorafenib (Nexavar®, a registered trademark of Bayer HealthCare Pharmaceuticals, Ltd., China) is an oral multikinase inhibitor of vascular endothelial growth factor receptor and platelet-derived growth factor receptor that inhibits tumor-cell proliferation and tumor angiogenesis [7, 8]. A number of studies examining the safety and efficacy of sorafenib in HCC, both alone, and in combination with conventional TACE, have been conducted globally [9–13], and specifically in the Asia-Pacific region [14–17]. The Sorafenib Hepatocellular Carcinoma Assessment Randomised Protocol (SHARP) trial, an international, multi-centered, phase III placebo-controlled study of 602 patients with advanced HCC, found that sorafenib significantly prolonged survival in patients with unresectable HCC, with an overall survival of 10.7 months compared to 7.9 months for placebo, with an acceptable safety profile [13]. The 2009 Asia-Pacific (AP) study showed that sorafenib was well tolerated and demonstrated a significant improvement in OS over placebo in the treatment of HCC in Asian patients [14].

The Global Investigation of therapeutic DEcisions in hepatocellular carcinoma and Of its treatment with sorafeNib (GIDEON) trial, a non-interventional surveillance study, evaluated the safety and efficacy of sorafenib in patients with unresectable HCC in real-world practice [18]. The GIDEON trial generated a large database of HCC patients (approximately 3000) across different disease subclasses and stages, in different regions. Two interim analyses [19, 20] reported global and regional differences in patient baseline characteristics, disease etiology, real-world practice patterns, and treatment outcomes. Data from China in the GIDEON database provides the ability to examine the effects of sorafenib in a Chinese population. Liu et al. [21] examined sorafenib and TACE in the GIDEON Chinese subgroup and found that sorafenib was usually administered in patients with tumor progression or poor liver function after TACE and that survival outcomes were still considered satisfactory. Ye et al. [22] also studied the GIDEON Chinese subgroup and reported that earlier administration of sorafenib may improve outcomes in patients with unresectable HCC and portal vein tumor thrombosis.

Sorafenib is typically administered at a dose of 400 mg twice a day; however, in practice dosing varies based on physician preference, Child-Pugh class, performance status, and comorbidities [23, 24]. Few studies have reported sorafenib efficacy and safety with respect to dosing and Child-Pugh class in HCC patients. Analysis of the GIDEON data indicated that the median overall survival (OS) was longer for Child-Pugh A patients (13.6 months) compared to Child-Pugh B patients (5.2 months), though the median time to progression (TTP) was similar (4.7 vs. 4.4 months, respectively) [25]. Though most patients received a starting dose of 800 mg/day, the median dose in Child-Pugh A and B patients was 680 mg/day and 721 mg/day respectively, and the median duration of administration in the 2 groups was 13.7 weeks vs. 8.6 weeks, respectively [20].

Daniele et al. [26] evaluated sorafenib dosing and safety in the European subset of GIDEON, and reported that the majority of patients were started on 800 mg/day, and that 800 and 400 mg/day doses had similar adverse event profiles. However, the incidence of adverse events (all grades), drug-related adverse events, and serious adverse events was lower in patients treated with 800 mg/day, with the exception of hand-foot skin reaction (HFSR), which was lower in the 400 mg/day group. Subgroup analysis of the AP trial indicated that sorafenib treatment improved outcomes of advanced HCC patients regardless of baseline status [27].

The purpose of the present analysis was to investigate the safety of sorafenib in Chinese patients with unresectable HCC in real-world practice. This analysis also evaluated dosing in China, as well as the safety and efficacy in patients with different Child-Pugh class.

## Methods

### Study design

Data from the GIDEON Chinese subgroup were analyzed retrospectively. All included patients provided signed informed consent to participate in the GIDEON trial. The protocol and documentation were approved by the relevant institutional review boards, ethics committees and health authorities (Supplemental Data).

### Patients

Out of a total of 345 Chinese patients with unresectable HCC identified in the GIDEON trial database, 331 were eligible for the current analysis. Eligible patients had a radiographic, histologic or cytologic diagnosis of unresectable HCC and had a life expectancy of at least 8 weeks [18]. Exclusion criteria were consistent with the locally approved sorafenib (Nexavar) product information [18]. All patient treatment decisions were determined solely at the treating physicians’ discretion.

### Sorafenib treatment

Indications for sorafenib were consistent with the local sorafenib package information. The dosage of sorafenib and duration of therapy were determined by treating physicians based on individual patient conditions. All included patients were treated with sorafenib at least once. The duration of observation was from the initiation of sorafenib to withdrawal from the study, final follow-up visit, or death. Patients were seen after initiating sorafenib, during therapy, and after completion of sorafenib therapy until withdrawal from the study, loss to follow-up, or death.

### Data collection

Data collection for the GIDEON study has been previously described in detail [18–20]. Patient information was collected using both paper and electronic case report forms. At first visit, detailed patient demographics, baseline disease characteristics, previous therapies and sorafenib starting dose were recorded. At each physician visit, the date of the visit, HCC evaluation findings, and patient condition (as previously described), sorafenib therapy information (i.e., current dose, dose adjustments, and clinical benefit), adverse events and serious adverse events, and concomitant use of other drugs and therapies for HCC were recorded. All patient information was protected, and comparisons of patient data were done anonymously.

### Definitions of adverse events and serious adverse events

For the purposes of this study, an adverse event was defined as any unfavorable or unintended symptom, sign, or disease (including abnormal laboratory results or state of mind) associated with the use of sorafenib. Such effects may or may not be intervention-related, dose-related, route-related, patient-related, or caused by an interaction with another drug, which must be specified in recording the adverse events. A serious adverse event was defined as an adverse event resulting in any of the following at any dose: death, life-threatening condition (i.e., high risk of death), hospitalization (except for observation, convalescence, or planned surgery), persistent or evident disability (impaired daily life), birth defects/congenital malformation, or other important medical events determined by clinicians.

### Study end points and safety and efficacy assessment

The primary objective of the current analysis was to evaluate the safety of sorafenib in patients with unresectable HCC in routine clinical practice. All adverse events and serious adverse events occurring in all patients at any time, related or non-related to sorafenib therapy, were recorded, including date of presence or absence of adverse events, adverse event grade, relationship between adverse events and sorafenib (treatment-related adverse events), and measures taken to relieve the adverse events and related outcomes. The clinical characteristics of adverse events and serious adverse events, and the time from beginning sorafenib to occurrence of the adverse event were recorded. Adverse events were graded using the National Cancer Institute Common Terminology Criteria version 3.0 [28].

Dosage, duration of treatment, dose reductions/interruptions (plus reasoning), Child-Pugh class, ECOG-PS, and liver function were all documented at baseline and/or follow-up. Efficacy end points included OS, progression-free survival (PFS), and time to progression (TTP) as defined by Response Evaluation Criteria In Solid Tumors [29].

### Statistical analysis

All statistical analyses were performed with SAS software version 9.3 (SAS Institute Inc., Cary, NC). Adverse events analysis was conducted based on data from the safety population, and efficacy analysis was conducted on the intention-to-treat population. Patient demographic data and baseline characteristics were presented as mean and standard deviation for continuous variables, and by count and percentage for categorical variables. Safety data, including adverse events, serious adverse events, and death were presented as count and percentage according to Child-Pugh class at the start of therapy. Data regarding study drug administration were summarized as mean and standard deviation and median (range) for continuous data, and count and percentage for categorical data. The associations between baseline disease characteristics with OS, PFS, and TTP were assessed by the Kaplan-Meier method.

## Results

### Patient characteristics at study entry

**Fig. 1:**
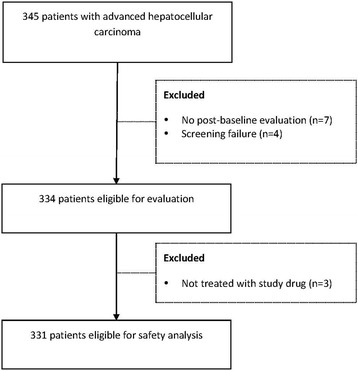
Flow diagram of patient disposition

Of the 345 Chinese patients in the database, 14 patients were excluded: seven did not attend any evaluations after enrollment, four failed screening, and three were not treated with the study drug. Thus, data of 331 eligible patients who met the inclusion criteria were included as the safety population (Fig. 1).

Patients demographic and baseline characteristics are summarized in Table 1. The mean age of the safety population was 50.0 ± 11.5 years (range, 19–85 years), the majority of patients were male (92%) and less than 65 years of age (90%). Among all patients, 89% had hepatitis B infection, 33% had more than three liver lesions, 43% had HCC confined to the liver, 40.5% had extrahepatic HCC, 30% had portal vein thrombosis, and 22% had vascular invasion. Approximately 39% of patients were tumor-node-metastasis (TNM) stage at III, and 22% were stage IV. Based on the Barcelona Clinic Liver Cancer (BCLC) staging classification, 23% were class A or B, and 67% of were class C or D. Majority of patients, were Child-Pugh A (67%) and 12% were Child-Pugh B or C. Prior to sorafenib treatment, 34% patients received surgery and 52% received TACE.

**Table 1:** Characteristics of the safety population (*N* = 331)

Age (years)	50.0 ± 11.5
< 65	297 (90)
65–75	25 (7.6)
≥ 75	7 (2.1)
Missing	2 (0.6)
Gender	
Male	305 (92)
Female	26 (7.9)
Hepatitis B	
Yes	296 (89)
No	6 (1.8)
Others^a^	36 (11)
Portal vein thrombosis	
Yes	98 (30)
No	190 (57)
Unknown	43 (13)
Number of liver lesions	
1–3	196 (59)
> 3	112 (34)
HCC confined to liver, yes	142 (43)
Vascular invasion, yes	74 (22)
Extrahepatic spread, yes	134 (41)
TNM stage	
I or II	60 (18)
III	129 (39)
IV	72 (22)
Missing or unknown	70 (21)
Barcelona Clinic Liver Cancer stage	
A or B	76 (23)
C or D	222 (67)
Not evaluable	33 (10)
Child-Pugh class	
A	220 (67)
B or C	38 (12)
Not evaluable	73 (22)
Cancer of the Liver Italian Program score	
0	49 (15)
1	51 (15)
2	54 (16)
3	51 (15)
4–6	46 (14)
Prior surgery	111 (34)
Prior locoregional therapy	
None	89 (27)
Percutaneous ethanol injection only	2 (0.6)
Radiofrequency ablation only	16 (4.8)
Transarterial chemoembolization only	172 (52)
Concomitant^b^	50 (15)
Others	2 (0.6)

Data are presented as mean ± standard deviation or count (percentage). Percentages are presented to 2 significant figs

*HCC* hepatocellular carcinoma, *TNM* tumor node metastasis

^a^Others include five hepatitis C, five alcohol use, 23 unknown, two other, and one other hepatobiliary disorders/findings

^b^Concomitant therapy refers to using at least two therapies for loco-regional anti-cancer treatment at baseline

### Sorafenib administration

Sorafenib administration of the overall study population is summarized in Table 2. Completed dosing data of 319 patients was available for review.. The median treatment duration was 22.3 weeks (range, 0.1–116.1 weeks); 41% of patients had a treatment duration > 28 weeks and only 6.3% had a treatment duration of ≤4 weeks. Overall, 98% of patients received an initial dose of 800 mg/day, and mean daily dose was 776.2 ± 83.6 mg/day (median daily dose 800 mg). Seven patients (2.1%) had dose interruptions, and five of these (71%) were due to adverse events. Twenty-seven patients (8.2%) required dose modification, and of these 16 (59%) were modified due to adverse events. Thirteen patients (3.9%) had a dose increase, and 21 (6.3%) required a dose reduction (Additional file 1: Table S1).

**Table 2:** Sorafenib administration in the safety population (*N* = 331)

Duration of treatment	
Missing	12 (3.6)
≤ 4 weeks	21 (6.3)
> 4 and ≤8 weeks	37 (11)
> 8 and ≤12 weeks	51 (15)
> 12 and ≤16 weeks	23 (6.9)
> 16 and ≤20 weeks	17 (5.1)
> 20 and ≤24 weeks	19 (5.7)
> 24 and ≤28 weeks	15 (4.5)
> 28 weeks	136 (41)
Treatment duration (weeks) for available patients	
Number	319
Mean ± SD	29.54 ± 24.79
Median (range)	22.30 (0.1 to 116.1)
Average daily dose (mg)	
Number	204
Mean ± S D	776.2 ± 83.6
Median (range)	800 (332 to 818)
Total dose (mg)	
Number	204
Mean ± SD	133,512.3 ± 115,060.0
Median (range)	198,350 (800 to 600,800)
Dose intensity (%)	
Number	204
Mean ± SD	96.5 ± 11.8
Median (range)	100 (26 to 102)
Actual days on study drug (excluding off days)	
Number	204
Mean ± SD	171.4 ± 146.3
Median (range)	127.5 (1 to 751)
Initial sorafenib dose level	
Number	331
400 mg	4 (1.2)
600 mg	1 (0.3)
800 mg	326 (98)
Total subjects with dose interruptions ticked, yes	7 (2.1)
Total subjects with dose modification ticked, yes	27 (8.2)
Total subjects with dose increase, yes	13 (3.9)
Total subjects with dose reduction, yes	21 (6.3)

Data are presented as mean ± standard deviation, median (range) or count (percentage). Percentages are presented to 2 significant figures

### Safety assessment

Safety data are reported in Tables 3, 4 and 5. Overall, 51% of patients experienced at least one treatment-emergent adverse event, and 6% were grade 3–4 adverse events. Drug-related adverse events were experienced by 29% of patients, and 3.6% had grade 3 or 4 events. Overall, 23% of patients (*n* = 77) experienced serious adverse events, and only one (0.3%) was drug-related (Table 3).

**Table 3:** Treatment-emergent adverse events by Child-Pugh class at start of therapy in the safety population

Adverse events	Total (*N* = 331)	Child-Pugh A (*n* = 246)	Child-Pugh B (*n* = 48)	Child-Pugh C (*n* = 2)	Not evaluable (*n* = 35)
Treatment-emergent AE (all grades including deaths not documented as AE)	233 (70)	175 (71)	38 (79)	0 (0)	20 (57)
AEs resulting in permanent discontinuation	29 (8.8)	22 (8.9)	5 (10)	0 (0)	2 (5.7)
Treatment-emergent AE (all grades)	167 (51)	124 (50)	27 (56)	0 (0)	16 (46)
Grade 3–4	20 (6.0)	14 (5.7)	4 (8.3)	0 (0)	20 (6.0)
Grade 5	73 (22)	55 (22)	11 (23)	0 (0)	7 (20)
Drug-related AE (all grades)	95 (29)	67 (27)	17 (35)	0 (0)	11 (31)
Grade 3–4	12 (3.6)	9 (3.7)	2 (4.2)	0 (0)	1 (2.9)
Grade 5	0 (0)	0 (0)	0 (0)	0 (0)	0 (0)
Serious AE (all grades)	77 (23)	58 (24)	12 (25)	0 (0)	7 (20)
Drug-related (all grades)	1 (0.3)	0 (0)	1 (2.1)	0 (0)	0 (0)
All deaths	166 (50)	127 (52)	27 (56)	0 (0)	12 (34)
Treatment-emergent death	73 (22)	55 (22)	11 (23)	0 (0)	7 (20)

Data are presented as count (percentage). Percentages are presented to 2 significant figs

*AE* adverse event, *SAE* serious adverse event

Treatment-emergent deaths: death while on sorafenib and up to 30 days after last dose collected from all available sources

All deaths: all deaths from all sources where death information was collected including follow-up information

**Table 4:** Incidence of treatment-emergent drug-related adverse events by worst grade and Child-Pugh class at start of therapy

Drug-related adverse events	Child-Pugh A	Child-Pugh B	Child-Pugh C
	(*n* = 246)	(*n* = 48)	(*n* = 2)
Any adverse event	67 (27)	17 (35)	0 (0)
Cardiac, general	8 (3.3)	1 (2.1)	0 (0)
Hypertension	7 (2.8)	1 (2.1)	0 (0)
Hypotension	2 (0.8)	0 (0)	0 (0)
Constitutional symptoms	2 (0.8)	0 (0)	0 (0)
Fatigue	1 (0.4)	0 (0)	0 (0)
Fever	1 (0.4)	0 (0)	0 (0)
Dermatology/skin	58 (24)	12 (25)	0 (0)
Alopecia	6 (2.4)	2 (4.2)	0 (0)
Hand-foot skin reaction	51 (21)	7 (15)	0 (0)
Rash/desquamation	8 (3.3)	3 (6.3)	0 (0)
Ulceration	0 (0)	0 (0)	0 (0)
Gastrointestinal	25 (10)	8 (17)	0 (0)
Diarrhea	25 (10)	7 (15)	0 (0)
Nausea	0 (0)	1 (2.1)	0 (0)

Data are presented as count (percentage). Percentages are presented to 2 significant figures

**Table 5:** Onset time for drug-related adverse events: diarrhea, hand-foot skin reaction, and liver dysfunction

Time of onset	Total	Child-Pugh A	Child-Pugh B	Not evaluable
Diarrhea	35/39	25/28	7/8	3/3
0–30 days	12/13	8/8	4/5	0/0
31–60 days	4/4	3/3	1/1	0/0
61–90 days	5/5	4/4	0/0	1/1
> 90 days	14/17	10/13	2/2	2/2
Hand-foot skin reaction	63/64	51/51	7/8	5/5
0–30 days	37/38	29/29	5/6	3/3
31–60 days	11/12	9/10	1/1	1/1
61–90 days	5/5	5/5	0/0	0/0
> 90 days	10/9	8/7	1/1	1/1
Liver dysfunction	0/46	0/30	0/9	0/7
0–30 days	0/6	0/2	0/2	0/2
31–60 days	0/2	0/1	0/1	0/0
61–90 days	0/10	0/8	0/1	0/1
> 90 days	0/28	0/19	0/5	0/4

Data are presented as represented as number of drug-related adverse events / number of adverse events

The incidence of adverse events and serious adverse events were broadly comparable between Child Pugh A and B patients. The majority of drug-related adverse events were grade 1 or 2 in Child-Pugh A and B patients, though the frequency of drug-related adverse events was greater in Child-Pugh B (35%) compared with Child-Pugh A (27%) patients (Table 3). The incidences of treatment-emergent adverse events by worst grade and Child-Pugh score at start of therapy are summarized in Additional file 1: Table S2.

The most frequent drug-related adverse events were comparable across Child-Pugh subgroups, and were consistent with those of the overall population (Table 4). Dermatological, HFSR, gastrointestinal, and diarrhea were the most frequently observed in both Child-Pugh A and B patients, with gastrointestinal adverse events slightly more frequent in Child-Pugh B than A patients (17% vs 10%).

**Fig. 2:**
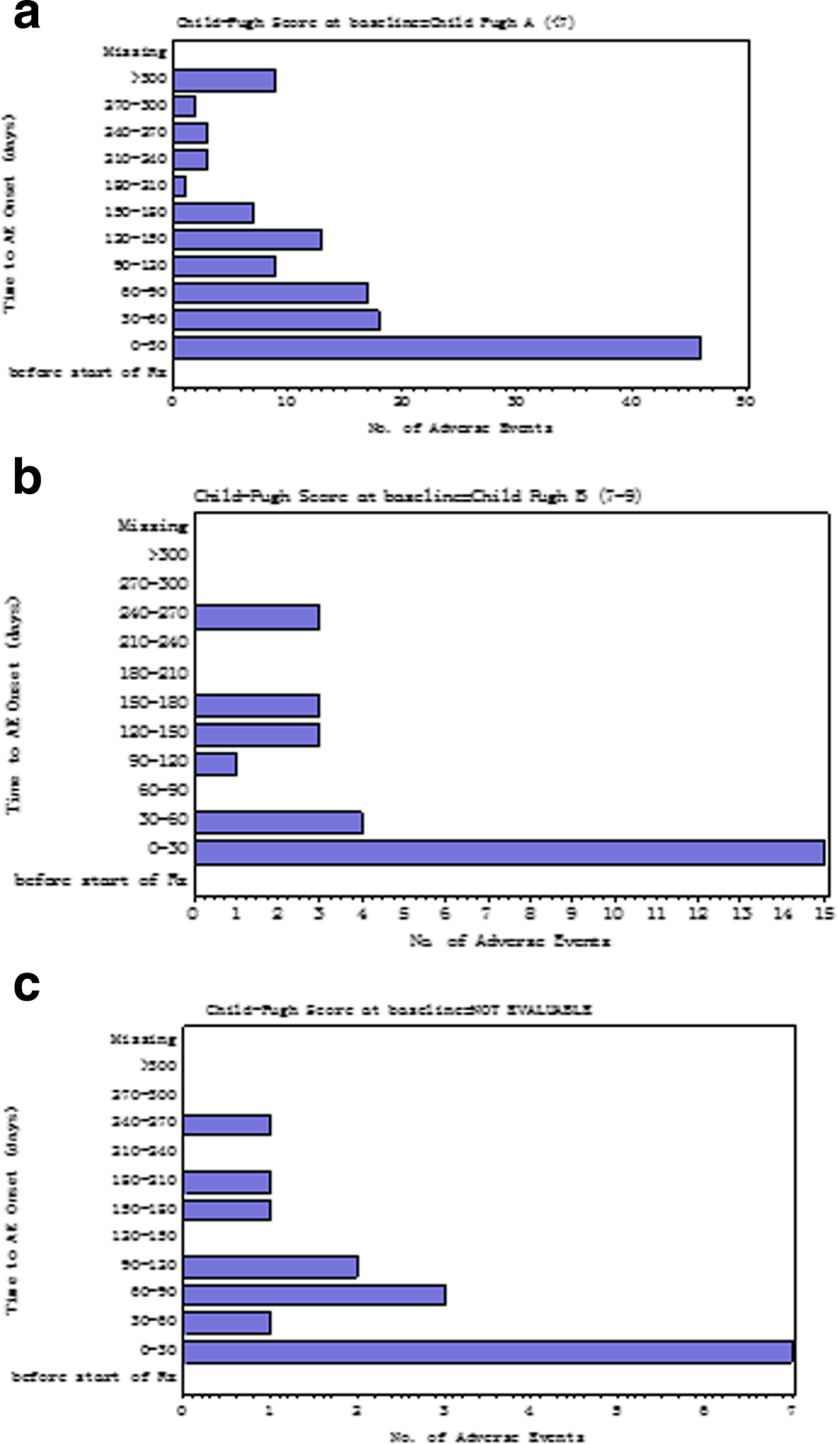
Time to adverse events onset after sorafenib administration in the safety population. **a** Child-Pugh class A (*n* = 246). **b** Child-Pugh class B (*n* = 38). **c** Child-Pugh class not evaluable (*n* = 35). Child-Pugh classes are those at baseline, minimum grade 1. AE: adverse events

The time to onset of all adverse events was within 30 days of starting sorafenib, then declined markedly (Fig. 2). However, the time to onset for specific adverse events varied (Table 5). Of 39 patients with diarrhea, 35 were drug-related. Onset of diarrhea most frequently occurred within 30 days, and after 90 days. Of 64 patients with HFSR, 63 were drug-related and the onset was typically within 30 days. Liver dysfunction occurred most frequently after 90 days with no drug-related cases reported.

### Efficacy (intention-to-treat population)

**Fig. 3:**
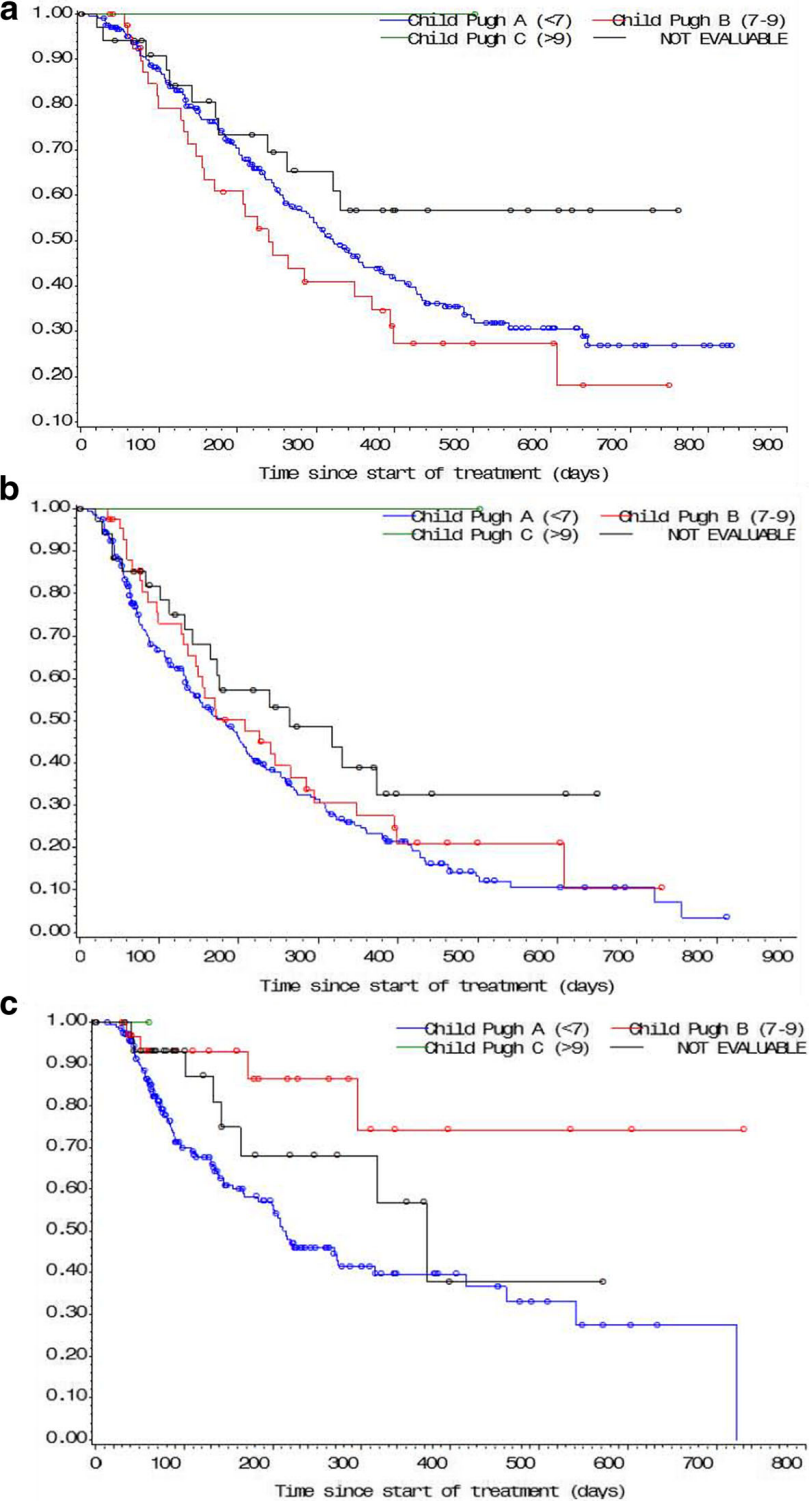
Kaplan-Meier curve survival for patients with unresectable HCC based on Child-Pugh score in the intention-to-treat population. **a** Overall survival (OS); (**b**) progression-free survival (PFS); (**c**) time-to-progression for Child-Pugh A versus Child-Pugh B, Child-Pugh C as defined by Response Evaluation Criteria in Solid Tumors criteria

Overall 378 patients were in interntion-to-treat population, there were 166 mortality events,222 progression events as defined by the Response Evaluation Criteria in Solid Tumors criteria. The median OS was 322 days (95% confidence interval [CI]: 270–382 days), the median PFS was 186 days (95% CI: 155–216 days), and median TTP was 274 days (95% CI: 209–463 days). The median OS was longer for Child-Pugh A patients compared to B (322 vs. 240 days), while the PFS time (183 vs. 208 days), and median TTP (214 days vs. not reached) were shorter for Child-Pugh A patients compared to Child-Pugh B patients (Fig. 3).

## Discussion

The majority of this Chinese subgroup was male (92%), more than 65 years old (90%), and had hepatitis B (89%). This population consisted of 67% Child-Pugh A patients, and 67% were BCLC stage C or D compared to the overall GIDEON safety population, where 61% of patients were Child-Pugh A and 21% were Child-Pugh B. In real-life clinical practice, over 98% of patients received the recommended initial sorafenib dose of 800 mg/day, with a median duration of treatment of 22.3 weeks (range, 0.1–116.1 months). In comparison to the overall GIDEON safety population,, where only 72% of Child-Pugh A and 70% Child-Pugh B patients received an initial sorafenib dose of 800 mg [30].

### Adverse events

About 50% of patients experienced at least one adverse event, with approximately 28% experiencing a drug-related adverse event. Majority of the drug-related adverse events were grade 1 or 2, which is consistent with what was reported in the GIDEON and Asia-Pacific phase III trials [13, 14]. The results are different from the SHARP trial in which 98% of sorafenib-treated patients had an adverse event, and 80% had a drug-related adverse event [23]. However, the incidence of serious adverse events was lower (23%) in the GIDEON Chinese subgroup compared with the serious adverse events incidence in the SHARP study (52%) [23].

In this subgroup, the most common drug-related adverse events were dermatology/skin, HFSR, gastrointestinal, and diarrhea which is similar to previous trials [14, 19, 23]. However, a very low percentage of patients reported having fatigue (0.4%) or rash (3.3% and 6.3% for patients with Child-Pugh A and Child-Pugh B, respectively), which differs from SHARP [23], AP [14], and GIDEON [19], where fatigue and rash/desquamation were some of the most commonly reported drug-related adverse events. In the AP study, the most common grade 3 and 4 drug-related adverse events in sorafenib-treated patients were HFSR, diarrhea, and fatigue, and fatigue was the most common in patients receiving placebo [27]. Al-Rajabi et al. [24] reported nausea in 70.8% of all patients, and diarrhea in 57.5%, and the percentages were similar between Child-Pugh A and B patients. In general, gastrointestinal distress (diarrhea) is a common adverse event associated with sorafenib, whether it is used for HCC or advanced renal cell carcinoma [31, 32]. Other studies of GIDEON Chinese subset have shown sorafenib is associated with adverse events regardless of prior surgery, the presence of portal vein tumor thrombosis [22], or with prior or concomitant TACE [21].

### Influence of child-Pugh class on safety

Overall, adverse events were comparable between Child-Pugh A and B patients, including the most frequently occurring drug-related adverse events. However, the Chinese subgroup differed from the overall global GIDEON cohort with regard to drug-related adverse events with more events occurring in Child-Pugh B (35%) vs. Child-Pugh A (27%) patients.. Additionally, the percentage of Chinese patients experiencing drug-related adverse events was much lower than reported for the overall GIDEON cohort in the second interim analysis (63% for Child-Pugh B; 67% for Child Pugh A) [20]. Toxicity in terms of all-grade adverse events was lower in Child-Pugh A (50%) and B (56%) patients. In addition, there were no differences between Child-Pugh A and B patients with respect to the percentage of deaths and treatment-emergent deaths. A serious drug-related adverse event was only reported in one Child-Pugh B patient (0.3%), and none were reported in Child-Pugh A patients. The two Child-Pugh C patients had no adverse events, serious adverse events, or death; however, this number is too small to make meaningful comparisons.

Differences in patient baseline clinical characteristics, in initial sorafenib dose and administration, and in the concomitant locoregional therapy may have contributed to differences between this study and others. In the present study, initial sorafenib dose of 800 mg per day was the most common dosage, which is consistent with most studies. Adverse events were the main reason for dose interruptions and drug discontinuation in the present study, and in other studies. Reasons for dose modification included adverse events, disease progression, and concomitant treatments/procedures (Additional file 1: Table S1). Our results suggest that a longer duration of sorafenib treatment with the recommended daily dose of 800 mg may contribute to greater clinical benefit in terms of survival with an acceptable safety profile.

### Influence of child-Pugh class on efficacy

In the present study the overall median OS was 322 days (10.7 months), compared with the median OS recorded in the Asia-pacific study (6.5 months for total AP population) [27]. It should be noted, however, that although our observed median OS was longer, in our population 22% and 41% of the patients had tumor burden, vascular invasion and extra-hepatic spread, respectively; as compared to 79% of the patients had either or both tumor burden in the Asian-pacific study. These comparisons suggest that patients with less advanced disease at the start of therapy may experience a greater survival benefit.

Final analysis of the GIDEON data showed the median OS was greater in Child-Pugh A patients (13.6 months, 95% CI: 12.8–14.7 months) than in B patients (5.2 months, 95% CI: 4.6–6.3 months) [25]. Consistent with the findings of a recent study by Al-Rajabi et al. [24], our population, with 11.5% Child-Pugh B patients, had a difference in median OS between Child-Pugh A and B (10.7 vs. 8 months, respectively). In the second interim analysis of the GIDEON data, most patients received the approved sorafenib dose of 800 mg/day, but the median duration of therapy was shorter in Child-Pugh B patients [20]. A recent European subgroup analysis of GIDEON data suggested that a higher starting dose may result in longer OS [26]. Liu et al. [21] reported that the Chinese subpopulation had more advanced HCC at study entry than the overall GIDEON population (BCLC C/D, 67% vs. 58%; TNM III/IV, 77% vs. 72%), but better underlying liver function (Child-Pugh B/C, 16% vs. 58%), and Chinese patients with the best liver function (Child-Pugh A) had a longer OS than GIDEON patients as a whole (13.6 months vs. 10.7 months), while those with worse liver function (Child-Pugh B) had a shorter OS (5.0 months vs. 8.0 months).

### Limitations

This study has certain limitations, including the retrospective analysis of data which limits evaluation of causality. The descriptive results should be interpreted with caution as statistical analysis for significance was not performed. Data were collected from multiple tertiary care centers in China, and the findings may not translate fully to daily practice in primary care settings. Whether patients were given continuous or interrupted sorafenib dosing was not studied. In addition, only 1.2% of patients received an initial dose of 400 mg, and thus the safety and efficacy of this dose could not be studied. Furthermore, only 12% of patients were Child-Pugh B or C, and among them there were only two evaluable Child-Pugh C patients. Similarly, the percentages of patients receiving a 400 mg (1.2%) and 600 mg (0.3%) starting dose were too small to allow a meaningful comparison. Lastly, we did not collect data or study the influence of hepatitis B viral load.

## Conclusions

This study has shown that sorafenib has acceptable efficacy and safety in Chinese Child-Pugh A and B patients with unresectable HCC using the recommended dosage of 800 mg/day, and that safety and the occurrence of adverse events are not affected by liver function. Different than seen in other phase III sorafenib trials, gastrointestinal adverse events were the most common drug-related adverse events in Chinese patients with unresectable HCC administered sorafenib. Taken together, these results suggest that sorafenib treatment is appropriate for Chinese patients with unresectable HCC.

## Additional file

Additional file 1: **Table S1.** Reasons for dose interruptions or dose modifications. **Table S2.** Incidences of treatment-emergent adverse events by worst grade and Child-Pugh score at start of therapy are summarized. (DOC 63 kb)

## Abbreviations

AE: adverse event
BCLC: Barcelona Clinic Liver Cancer
CI: confidence interval
CT: computed tomography
ECOG-PS: Eastern Cooperative Oncology Group performance status
GIDEON: Global Investigation of therapeutic DEcisions in hepatocellular carcinoma and Of its treatment with sorafeNib
HCC: hepatocellular carcinoma
HFSR: hand-foot skin reaction
OS: overall survival
PFS: progression-free survival
SAE: serious adverse event
SD: standard deviation
SHARP: Sorafenib Hepatocellular Carcinoma Assessment Randomised Protocol trial
TACE: transarterial chemo-embolization
TNM: tumor-node-metastasis
TTP: time to progression
