# Supplementary material for: Safety assessment of sorafenib in Chinese patients with unresectable hepatocellular carcinoma: subgroup analysis of the GIDEON study
Source: BMC Cancer. 2018 Mar 2;18:247. doi: 10.1186/s12885-018-4144-9 (PMC5834849; doi:10.1186/s12885-018-4144-9)
Supplement: Supplementary file 1 — Table S1. Reasons for dose interruptions or dose modifications. Table S2. Incidences of treatment-emergent adverse events by worst grade and Child-Pugh score at start of therapy are summarized. (DOC 63 kb) [file 12885_2018_4144_MOESM1_ESM.doc]

**Additional file 1**

**Table S1. Reasons for dose interruptions or dose modifications (N=331)**

| **Reason for dose interruption** | **n=7** |
| --- | --- |
| Adverse event(s) | 5 |
| Economic reasons/drug shortage | 1 |
| Surgical treatment | 1 |
| **Reason for dose modification** | **n=19** |
| Adverse events | 16 |
| Disease progression | 1 |
| Concomitant treatment/procedure | 1 |
| Confirmed tolerability | 1 |
| Unknown | 1 |

**Table S 2. Incidence of treatment-emergent adverse events by worst grade and Child-Pugh class at start of therapy**

| **Treatment-emergent adverse events** | **Child-Pugh A** | **Child-Pugh B** | **Child-Pugh C** |
| --- | --- | --- | --- |
|  | **(n=246)** | **(n=48)** | **(n=2)** |
| Dermatology/skin | 58 (24) | 13 (27) | 0 (0) |
| Alopecia | 7 (2.8) | 2 (4.2) | 0 (0) |
| Hand-foot skin reaction | 51 (21) | 8 (17) | 0 (0) |
| Rash/desquamation | 8 (3.3) | 3 (6.3) | 0 (0) |
| Ulceration | 0 (0) | 0 (0) | 0 (0) |
| Gastrointestinal | 31 (13) | 9 (19) | 0 (0) |
| Anorexia | 2 (0.8) | 1 (2.1) | 0 (0) |
| Diarrhea | 28 (11) | 8 (17) | 0 (0) |
| Distension | 0 (0) | 1 (2.1) | 0 (0) |
| Mucositis (functional/symptomatic), small bowel | 1 (0.4) | 0 (0) | 0 (0) |
| Nausea | 1 (0.4) | 1 (2.1) | 0 (0) |
| Gastrointestinal obstruction | 1 (0.4) | 0 (0) | 0 (0) |
| Hemorrhage/bleeding | 16 (6.5) | 2 (4.2) | 0 (0) |
| Hemorrhage with surgery | 1 (0.4) | 0 (0) | 0 (0) |
| Hemorrhage, gastrointestinal, liver | 1 (0.4) | 0 (0) | 0 (0) |
| Hemorrhage, gastrointestinal, oral cavity | 0 (0) | 1 (2.1) | 0 (0) |
| Hemorrhage, gastrointestinal, stomach | 0 (0) | 1 (2.1) | 0 (0) |
| Hemorrhage, gastrointestinal, upper gastrointestinal, non-specified | 14 (5.7) | 0 (0) | 0 (0) |
| Hepatobiliary/pancreas | 30 (12) | 9 (19) | 0 (0) |
| Liver dysfunction | 30 (12) | 9 (19) | 0 (0) |
| Metabolic/laboratory | 1 (0.4) | 1 (2.1) | 0 (0) |
| Alanine aminotransferase | 1 (0.4) | 0 (0) | 0 (0) |
| Bilirubin | 0 (0) | 1 (2.1) | 0 (0) |
| Metabolic/laboratory-other | 0 (0) | 0 (0) | 0 (0) |

Data are presented as count (percentage ).Percentages are presented to 2 significant figures.
